# Beta-Adrenoceptor Stimulation Reveals Ca^2+^ Waves and Sarcoplasmic Reticulum Ca^2+^ Depletion in Left Ventricular Cardiomyocytes from Post-Infarction Rats with and without Heart Failure

**DOI:** 10.1371/journal.pone.0153887

**Published:** 2016-04-20

**Authors:** Mani Sadredini, Tore Kristian Danielsen, Jan Magnus Aronsen, Ravinea Manotheepan, Karina Hougen, Ivar Sjaastad, Mathis Korseberg Stokke

**Affiliations:** 1 Institute for Experimental Medical Research, Oslo University Hospital and University of Oslo, Oslo, Norway; 2 KG Jebsen Cardiac Research Center and Center for Heart Failure Research, University of Oslo, Oslo, Norway; 3 Bjørknes College, Oslo, Norway; 4 Clinic for Internal Medicine, Lovisenberg Diakonale Hospital, Oslo, Norway; Baylor College of Medicine, UNITED STATES

## Abstract

Abnormal cellular Ca^2+^ handling contributes to both contractile dysfunction and arrhythmias in heart failure. Reduced Ca^2+^ transient amplitude due to decreased sarcoplasmic reticulum Ca^2+^ content is a common finding in heart failure models. However, heart failure models also show increased propensity for diastolic Ca^2+^ release events which occur when sarcoplasmic reticulum Ca^2+^ content exceeds a certain threshold level. Such Ca^2+^ release events can initiate arrhythmias. In this study we aimed to investigate if both of these aspects of altered Ca^2+^ homeostasis could be found in left ventricular cardiomyocytes from rats with different states of cardiac function six weeks after myocardial infarction when compared to sham-operated controls. Video edge-detection, whole-cell Ca^2+^ imaging and confocal line-scan imaging were used to investigate cardiomyocyte contractile properties, Ca^2+^ transients and Ca^2+^ waves. In baseline conditions, i.e. without beta-adrenoceptor stimulation, cardiomyocytes from rats with large myocardial infarction, but without heart failure, did not differ from sham-operated animals in any of these aspects of cellular function. However, when exposed to beta-adrenoceptor stimulation, cardiomyocytes from both non-failing and failing rat hearts showed decreased sarcoplasmic reticulum Ca^2+^ content, decreased Ca^2+^ transient amplitude, and increased frequency of Ca^2+^ waves. These results are in line with a decreased threshold for diastolic Ca^2+^ release established by other studies. In the present study, factors that might contribute to a lower threshold for diastolic Ca^2+^ release were increased THR286 phosphorylation of Ca^2+^/calmodulin-dependent protein kinase II and increased protein phosphatase 1 abundance. In conclusion, this study demonstrates both decreased sarcoplasmic reticulum Ca^2+^ content and increased propensity for diastolic Ca^2+^ release events in ventricular cardiomyocytes from rats with heart failure after myocardial infarction, and that these phenomena are also found in rats with large myocardial infarctions without heart failure development. Importantly, beta-adrenoceptor stimulation is necessary to reveal these perturbations in Ca^2+^ handling after a myocardial infarction.

## Introduction

Contractile dysfunction is the key feature of heart failure, but arrhythmias are an equally important cause of increased mortality from this condition [1]. Altered Ca^2+^ handling contributes importantly to the pathophysiology, and many models of heart failure exhibit decreased Ca^2+^ transient amplitude in ventricular myocytes [2–7]. This is often explained by decreased sarcoplasmic reticulum (SR) Ca^2+^ content that results from a combination of decreased SR Ca^2+^ reuptake and increased SR Ca^2+^ leak [8]. However, just as commonly, ventricular myocytes from animals with heart failure have been found to have increased propensity for abnormal SR Ca^2+^ release events associated with arrhythmias [9, 10]. These Ca^2+^ release events occur when SR Ca^2+^ content exceeds a certain threshold level [11, 12]. This threshold level is modified by expression and activity of Ca^2+^ handling proteins, with the SR Ca^2+^ release channel, ryanodine receptor type 2 (RyR2), playing a key role [12, 13]. Although the detailed molecular mechanisms are still being intensely investigated, post-translational modification of RyR is well established as a major factor in setting the threshold level [14–16], with most attention given to the downstream effects of beta-adrenoceptor stimulation through Ca^2+^/calmodulin-dependent protein kinase II (CaMKII) and protein kinase A [14, 17–19].

The accumulated insight in the processes underlying contractile dysfunction and arrhythmias in heart failure come from a number of models, both in terms of species and interventions to induce heart failure. However, most studies of heart failure have focused on either Ca^2+^ homeostasis related to contractility *or* abnormal Ca^2+^ release events associated with arrhythmias. Fewer studies show that these features really coexist in clinically relevant models of heart failure. Experimental studies of heart failure are mostly based on animal models of end-stage heart failure, and rarely on clinically relevant intermediate forms of the disease. In addition, descriptions of heart failure after myocardial infarction, the most common cause of heart failure, are mostly based on comparisons to sham-operated animals, rather than the equally clinically relevant post-infarction animals without heart failure. The aim of this study was therefore to investigate whether increased diastolic Ca^2+^ release events and reduced SR Ca^2+^ content could be found in ventricular cardiomyocytes from a well-characterized rat model of myocardial infarction from which two groups with distinct phenotypes can be distinguished, i.e. rats with developing heart failure and rats with myocardial infarction but without heart failure.

## Methods

### Ethics statement

Animal experiments were approved by the Norwegian National Animal Research Authority (FOTS ID 4173 and 6577), which conforms to the National Institute of Health guidelines (NIH publication No. 85–23, revised 1996) and European Directive 2010/63/EU. Two animals per cage were housed in a temperature-regulated room with a 12:12 h light-dark cycle, and unlimited access to food and water. Euthanasia was performed by cardiac excision or cervical dislocation during anesthesia with 4.5% isofluorane inhalation. Criteria for humane endpoints was a) acute decompensated heart failure (reduced mobility of the animal combined with piloerection, respiratory stridor, peripheral edema with swollen eyelids), b) postoperative wound infection (substantial pus from the wound) and c) major disruption of the surgical wound.

### Surgical procedure

Male Wistar rats (~300 g) were anesthetized in a chamber with a mixture of O_2_ and 4.5% isoflurane (Abbott Laboratories), and subsequently ventilated through endotracheal intubation on a Zoovent ventilator (Triumph Technical Services, Milton Keynes, UK) with 68% N_2_O, 29% O_2_, and 2% isoflurane. Thoracotomy was performed on the left side, the heart was exteriorized and the left coronary artery was ligated by a silk suture inducing a large myocardial infarction as described elsewhere [20]. The same surgical procedure was performed on the sham-operated animals, but the left coronary artery was not ligated. For postoperative analgesia, buprenorphine (0.2 mg/kg) was administered subcutaneously immediately after the surgical procedure.

### Echocardiography

Echocardiography was performed with a Vevo 2100 (VisualSonics, Ontario, Canada) on anesthetized rats, six weeks after coronary artery ligation. Data were collected from parasternal long and short axis images of the left ventricle and left atrium, Doppler signals in the mitral, pulmonary and aortic valves and tissue velocity in the left ventricular posterior wall as previously described [21]. Fractional shortening (FS) was calculated with the following formula: (LVDd-LVDs)/LVDd (LVDd, left ventricular diameter in diastole, LVDs, left ventricular diameter in systole). Furthermore, cardiac output (CO) was calculated with the following formula: heart rate x VTI_LVOT_ x πr_aorta_^2^ (VTI, velocity time integral, LVOT, left ventricular outflow tract).

### Group stratification

Post-myocardial infarction rats were stratified into two groups: rats with developing congestive heart failure (CHF) and rats with large myocardial infarction (> 30%) but without heart failure development (MI). The inclusion criteria for MI were lung weight under 2.5 g and left atrial diameter (LAD) under 5.0 mm, and for CHF lung weight over 2.5 g and LAD over 5.0 mm [22]. The sham-operated animals (Sham) served as controls.

### Cell isolation

Six weeks after coronary artery ligation, animals were weighed, intubated and ventilated as described under *surgical procedure*. A large thoracotomy was carried out; 200 IU heparin was administered intravenously through the hepatic vein before the heart was excised and immediately put in ice-cold saline (0.9% NaCl, B. Braun, Melsungen, Germany). The heart and lungs were weighed. The heart was then mounted on a modified Langendorff setup with cannulation of the aorta, and retrogradely perfused with ~30 ml 37°C oxygenated solution A: 130 mM NaCl, 25 mM N-2-hydroxyethylpiperazine-N'-2-ethanesulfonic acid (HEPES), 22 mM Glucose monohydrate, 5.4 mM KCl, 0.5 mM MgCl_2_, 0.4 mM NaH_2_PO_4_ (pH 7.4). The heart was then perfused with 37°C oxygenated solution A supplemented with ~200 U/ml collagenase Type-II (Worthington, NJ) and 0.08 mM Ca^2+^ for 20 min. An incision was made in viable myocardium to assure that the border zone proximal to the infarction was excluded. In Sham, cardiomyocytes were harvested from both the septum and the left ventricular free wall. The viable left ventricle free wall and septum were chopped into small pieces and gently stirred in solution A supplemented with 15 μM bovine serum albumin (Sigma Aldrich) and ~100 U/mL deoxyribonuclease I (Worthington, NJ). This mixture was transferred to a centrifuge tube through a Nitex mesh with 255 μm openings (Sefar). Cardiomyocytes sedimented by gravity were transferred to solution A supplemented with 0.1 mM Ca^2+^ and 15 μM bovine serum albumin. After subsequent sedimentations the cardiomyocytes were transferred to solution A supplemented with 0.2 mM Ca^2+^ and 15 μM bovine serum albumin, and finally 0.5 mM Ca^2+^ and 7.5 μM bovine serum albumin. Cardiomyocytes were stored at room temperature and used within 10 h of isolation.

### Cellular experiments

Cardiomyocytes were loaded with 5 μM fluo-4 AM (Molecular Probes), 10 min for line-scan images and 15 min for whole-cell fluorescence, and plated on laminin-coated coverslips mounted in a perfusion chamber. To allow sufficient time for the cell to attach to the cover slip and the de-esterification of the fluorescent probes, superfusion was only started after five minutes. Cardiomyocytes were superfused with solution B: 140 mM NaCl, 5.4 KCl, 0.5 mM MgCl_2_, 1 mM CaCl_2_, 5.5 glucose, 0.4 mM NaH_2_PO_4_, 5 mM HEPES (pH 7.4, preheated to 37 ± 0.1°C). Another 1 min was allowed for de-esterification as field-stimulation was applied with platinum electrodes at ~20% above the individual threshold for contraction for 30 s at 1 Hz followed by a period of 20 s without stimulation. For measurements of SR Ca^2+^ content, stimulation was applied for another 30 s before electrical stimulation was stopped with a simultaneous switch to solution B supplemented with 10 mM caffeine (Sigma Aldrich). The protocol was repeated after 100 s exposure to 20 nM isoprenaline (Norwegian Pharmacy Association) added to solution B and 1 Hz electrical stimulation. Ca^2+^ transients, Ca^2+^ waves, Ca^2+^ sparks and caffeine-induced Ca^2+^ release were recorded using line-scan imaging (Zeiss LSM *7 Live* confocal microscope) and whole-cell Ca^2+^ imaging (PhotoMed PTI microscope Photometer D-104G). Only cells without visible Ca^2+^ waves for 10 s before electrical stimulation, with preserved striation and no blebs were included. Cell shortening was recorded in Clampex software (version 9.0.0.710, Axon Instruments, Inc.) with a Nikon Diaphoto-TMD inverted microscope and a video edge detector (Crescent Electronics, Sandy, UT, USA).

### Immunoblotting

Hearts were excised as described under *Cell isolation* and the viable left ventricle including ventricular septum was rapidly frozen using liquid nitrogen. Frozen ventricular tissue was homogenized in buffer (210 mM sucrose, 2mM EGTA, 40 mM NaCl, 30 mM HEPES, 5 mM EDTA). Protease inhibitor, phosphatase inhibitor (Roche Diagnostics, Oslo, Norway) and sodium dodecyl sulfate (final concentration of 1%) was added. Micro Bicinchoninic Acid Protein Assay Kit (Thermo Fisher Scientific Inc., Rockford, IL) was used to quantify protein concentrations. The following antibodies were used (Table A in S1 File): Anti-CaMKIIδ ([23]), Anti-CaMKII Phospho Thr-286 (ab32678) (Abcam plc., Cambridge, UK), Anti-PP2A (Cat#05–421) (Millipore, Oslo, Norway), anti-RyR2 Phospho Ser-2808 (A010-30), anti-RyR2 Phospho Ser-2814 (A010-31), anti-Phospholamban Phospho Ser-16 (A010-12), anti-Phospholamban Phospho Thr-17 (A010-13) (Badrilla Ltd, Leeds, UK), Phospholamban (MA3-922), SERCA2 ATPase (MA3-919), anti-cardiac NCX (GenScript), RyR (MA3-916) (Thermo Fisher Scientific Inc.), GAPDH (Sc-20357), PP1 Antibody (E-9) (sc-7482) (Santa Cruz Biotechnology). Secondary antibodies: anti-goat IgG horseradish peroxidase conjugated antibody (R&D Systems, Oxon, UK), anti-rabbit or anti-mouse IgG horseradish peroxidase conjugated whole antibody (GE Healthcare, Oslo, Norway). 20 μg protein homogenate per lane was size fractionated on 4–15% Criterion TGX gels (Bio-Rad Laboratories, Oslo, Norway) and transferred to 0.45 μM PVDF-membranes (GE Healthcare). Membranes were then blocked in 5% non-fat milk or 1% Western Blocking Reagent (Roche Diagnostics) in Tris-buffered saline with 0.1% Tween (TBS-T) at room temperature for one hour. Primary antibody incubation was performed at 4°C overnight and secondary antibody incubation at room temperature for one hour. Enhanced Chemiluminescence (ECL Prime, GE Healthcare) was used to develop blots and signals were detected by LAS4000 (GE Healthcare). ImageQuant software (GE Healthcare) was used to quantify signals.

### Data analysis

Ca^2+^ transients, Ca^2+^ wave frequency and caffeine-induced Ca^2+^ releases were analyzed using Clampfit (version 9.0.0.710, Axon Instruments, Inc.). Ca^2+^ sparks were analyzed using ImageJ (version 1.45s, Wayne Rasband, National Institutes of Health, USA) and CaSparks (Daniel Ursu, 2003, Germany) [24]. Ca^2+^ wave velocities were analyzed using ImageJ. Data were expressed as mean ± standard error of the mean. Two-tailed distribution t-tests were performed in Microsoft Excel. Due to a skewed distribution of Ca^2+^ spark frequencies, these data were analyzed with a Poisson test using R software (version 3.0.2, The R Foundation for Statistical Computing). p<0.05 was considered statistically significant.

## Results

### 1. *In vivo* animal characteristics

**Fig 1:**
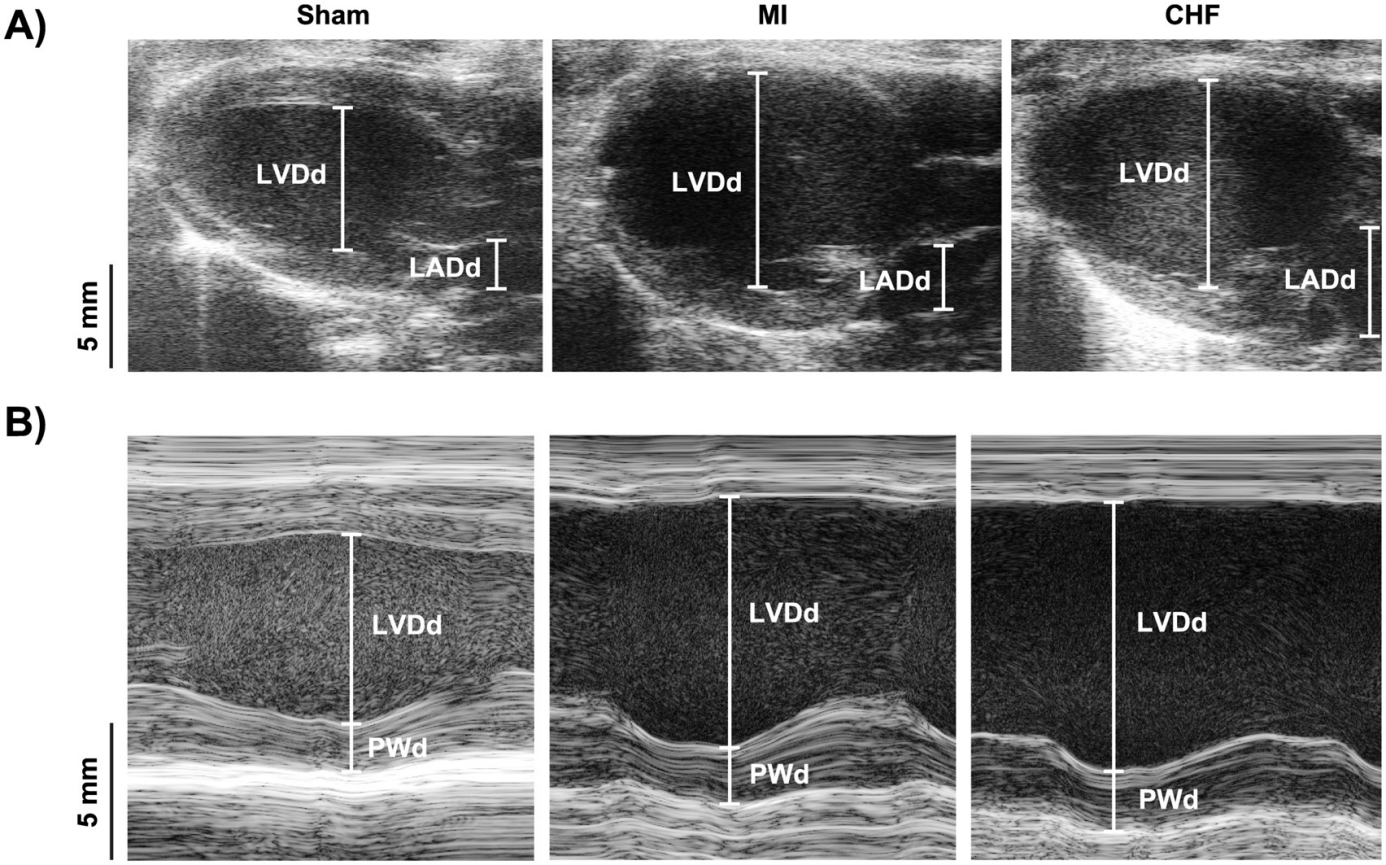
Echocardiographic measurements. Representative echocardiographic parasternal long axis (A) and m-mode (B) images. LVDd, left ventricular diameter in diastole, LADd, left atrial diameter in diastole, PWd, posterior wall thickness in diastole.

Post-myocardial infarction rats were stratified to CHF and MI groups based on echocardiographic criteria and lung weight. Representative echocardiographic images are shown in Fig 1. LAD was increased in both CHF and MI compared to Sham (Table 1). LAD value of 5.0 mm was used to distinguish between CHF and MI, consequently LAD was increased in CHF compared to MI. LVDd and LVDs, as well as FS were increased in both CHF and MI compared to Sham. However, posterior wall thickness in diastole (PWd) was only found to be increased in MI compared to Sham. Furthermore, heart rate was decreased in CHF compared to both Sham and MI. Increased peak mitral flow was found in MI, but not in CHF compared to Sham. Mitral deceleration was increased in both MI and CHF compared to Sham. No alteration in right ventricular outflow tract (RVOT) peak flow was observed in any of the groups. However, CO was decreased in CHF compared to Sham. Tissue Doppler measurements showed decreased maximal velocity in both CHF and MI compared to Sham, and in CHF compared to MI. Yet, no change in minimal velocity was observed in either group. Upon sacrifice, CHF rats with classic signs of heart failure had an increased lung weight and heart weight compared to Sham as sign of edema, pulmonary congestion and cardiac hypertrophy.

**Table 1:** *In vivo* animal characteristics.

	Sham	MI	CHF
**Animal data**			
Body weight (g)	414 ± 6	425 ± 12	435 ± 29
Heart weight (g)	1.5 ± 0.1	2.1 ± 0.2 *	2.8 ± 0.2 *
Heart weight normalized to body weight	0.0036 ± 0.0003	0.0049 ± 0.0004 *	0.0065 ± 0.0005 *
Lung weight (g)	1.6 ± 0.1	1.9 ± 0.1 *	4.3 ± 0.4 *,^#^
Lung weight normalized to body weight	0.0038 ± 0.0003	0.0045 ± 0.0002 *	0.0100 ± 0.0008 *,^#^
**Echocardiography**			
*M-mode*			
LAD (mm)	3.7 ± 0.1	4.3 ± 0.1 *	6.8 ± 0.3 *,^#^
LVDd (mm)	7.0 ± 0.1	9.4 ± 0.3 *	9.6 ± 0.3 *
LVDs (mm)	3.9 ± 0.1	7.8 ± 0.4 *	8.5 ± 0.4 *
FS (%)	44 ± 2	17 ± 2 *	12 ± 2 *
PWd (mm)	1.6 ± 0.1	2.0 ± 0.1 *	1.9 ± 0.2
*Doppler*			
Peak mitral flow (m/s)	0.89 ± 0.05	1.05 ± 0.03 *	1.09 ± 0.08
Mitral deceleration (m/s^2^)	28 ± 2	36 ± 3 *	47 ± 5 *
Peak RVOT flow (m/s)	0.77 ± 0.06	0.75 ± 0.05	0.62 ± 0.05
Heart rate (beats/min)	408 ± 12	410 ± 12	359 ± 17 *,^#^
CO in LVOT (ml/min)	123 ± 17	92 ± 15	65 ± 9 *
*Tissue Doppler*			
Maximal velocity (mm/s)	65 ± 4	51 ± 4 *	34 ± 4 *,^#^
Minimal velocity (mm/s)	-63 ± 5	-59 ± 1	-48 ± 10

LAD, left atrial diameter, LVDd, left ventricular diameter in diastole, LVDs, left ventricular diameter in systole, FS, fractional shortening, PWd, posterior wall thickness in diastole, RVOT, right ventricular outflow tract, LVOT, left ventricular outflow tract, CO, cardiac output,

*, p<0.05 vs. Sham,

^#^, p<0.05 vs. MI. 6–10 animals in each group.

### 2. Cardiomyocyte Ca^2+^ handling, contractile properties and arrhythmia-associated Ca^2+^ release events in absence of beta-adrenoceptor stimulation

**Fig 2:**
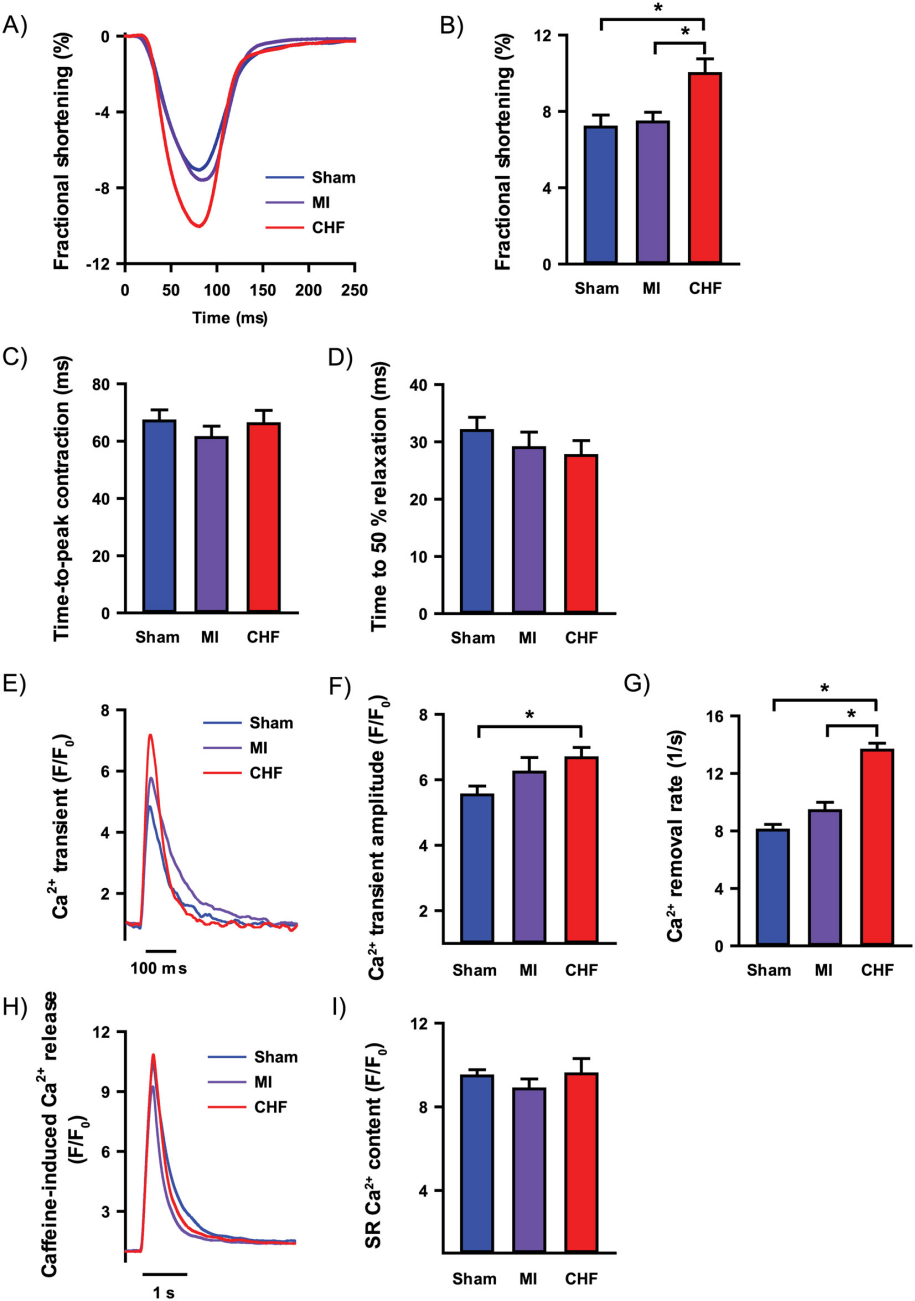
Cardiomyocyte contractile properties and Ca^2+^ handling. Recording of cardiomyocyte contraction cycle at 1 Hz field stimulation (A) with fractional shortening (B), time to peak contraction (C) and time to 50% relaxation (D). Ca^2+^ transients recorded at 1 Hz field stimulation using whole-cell Ca^2+^ imaging (E) with Ca^2+^ transient amplitude (F) and Ca^2+^ removal rate (G). Whole-cell Ca^2+^ imaging of caffeine-induced SR Ca^2+^ release (H) and SR Ca^2+^ content (I). n_heart_ = 2–7; n_cell_ = 14–67 for all analysis. *p<0.05 (T-test).

Cardiomyocyte Ca^2+^ handling and contractile properties were first studied in baseline conditions (Fig 2), i.e. without beta-adrenoceptor stimulation. Isolated ventricular myocytes from CHF rats showed increased fractional shortening compared to Sham (10.0 ± 0.8 vs. 7.2 ± 0.6%, p<0.05, Fig 2A and 2B). However, no alterations in time-to-peak contraction or time-to-half relaxation were found in ventricular myocytes from CHF compared to Sham (66.1 ± 4.7 vs. 67.1 ± 3.9 ms and 27.6 ± 2.6 vs. 32.0 ± 2.3 ms respectively, Fig 2C and 2D). Furthermore, CHF showed increased Ca^2+^ transient amplitude compared to Sham (F/F_0_: 6.7 ± 0.3 vs. 5.5 ± 0.3, p<0.05, Fig 2E and 2F). The CHF group also exhibited faster rate of Ca^2+^ removal compared to Sham (13.6 ± 0.5 vs. 8.1 ± 0.4 /s, p<0.05, Fig 2G), but no alterations in SR Ca^2+^ content (F/F_0_: 9.6 ± 0.7 vs. 9.5 ± 0.3, Fig 2H and 2I). MI cardiomyocytes did not differ from Sham in fractional shortening (7.5 ± 0.5 vs. 7.2 ± 0.6%, Fig 2B), time-to-peak contraction (61.3 ± 3.9 vs. 67.1 ± 3.9 ms, Fig 2C), time-to-half relaxation (29.0 ± 2.7 vs. 32.0 ± 2.3 ms, Fig 2D), Ca^2+^ transient amplitude (F/F_0_: 6.2 ± 0.4 vs. 5.5 ± 0.3, Fig 2F), rate of Ca^2+^ removal (9.4 ± 0.6 vs. 8.1 ± 0.4 /s, Fig 2G) or SR Ca^2+^ content (F/F_0_: 8.9 ± 0.5 vs. 9.5 ± 0.3, Fig 2H and 2I).

**Fig 3:**
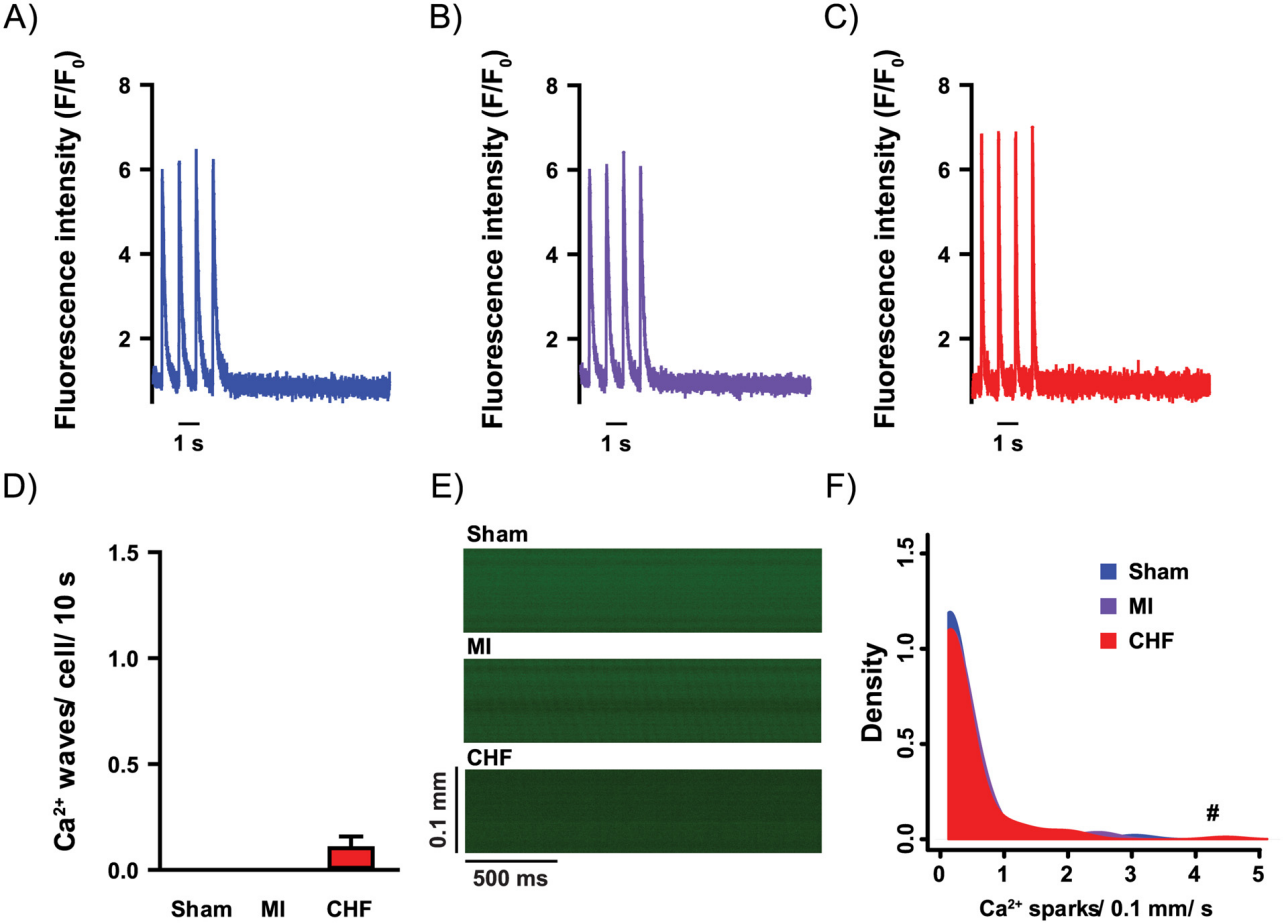
Cardiomyocyte Ca^2+^ release events. Whole-cell Ca^2+^ imaging was used to record Ca^2+^ transients in field-stimulated cardiomyocytes and a post-stimulation rest period of 10 seconds was recorded to analyze Ca^2+^ wave frequency in Sham (A), MI (B) and CHF (C). Few Ca^2+^ waves (D) were present under baseline conditions. Confocal line-scan recordings were used to analyze Ca^2+^ sparks (E). Increased Ca^2+^ spark frequency was found in CHF (F). n_heart_ = 3–7; n_cell_ = 18–98. ^#^p<0.05 (poisson test).

In absence of beta-adrenergic stimulation, no changes in Ca^2+^ wave frequency were found in ventricular myocytes from CHF or MI compared to Sham (Fig 3A–3D). Ca^2+^ spark frequency was increased in CHF myocytes compared to Sham (0.23 ± 0.06 vs. 0.13 ± 0.05 sparks/ 0.1 mm/ s, p<0.05, Fig 3E and 3F), but not in MI rats compared to Sham (0.20 ± 0.08 vs. 0.13 ± 0.05 sparks/ 0.1 mm/ s, Fig 3E and 3F). Thus, at baseline conditions, CHF rats with clinically evident heart failure, did not reveal reduced fractional shortening, reduced Ca^2+^ transient amplitude or increased occurrence of Ca^2+^ waves. Notably, MI rats without heart failure did not differ from Sham in either of these parameters of Ca^2+^ handling.

### 3. Cardiomyocyte Ca^2+^ handling, contractile properties and arrhythmia-associated Ca^2+^ release events during beta-adrenoceptor stimulation

**Fig 4:**
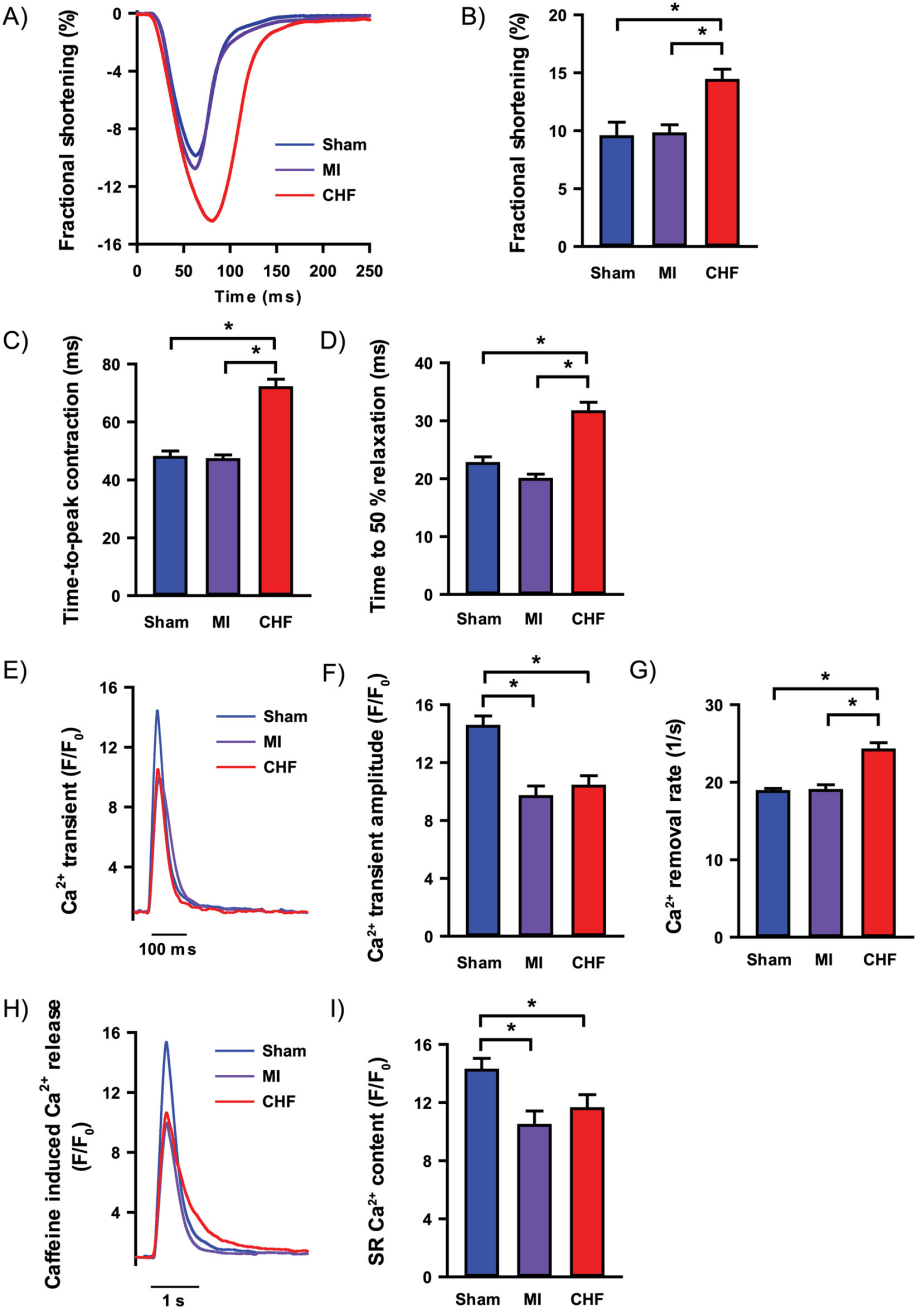
Cardiomyocyte contractile properties and Ca^2+^ handling under beta-adrenoceptor stimulation. Cardiomyocytes were subjected to 20 nM ISO to evaluate the effects of beta-adrenoceptor stimulation. Cardiomyocyte contraction cycle (A) with fractional shortening (B), time to peak contraction (C) and time to 50% relaxation (D). Ca^2+^ transients recorded using whole-cell Ca^2+^ imaging (E) with Ca^2+^ transient amplitude (F) and Ca^2+^ removal rate (G). Whole-cell Ca^2+^ imaging of caffeine-induced SR Ca^2+^ release (H) and SR Ca^2+^ content (I). n_heart_ = 2–6; n_cell_ = 10–29 for all analysis. *p<0.05 (T-test).

To simulate beta-adrenoceptor stimulation, myocytes were exposed to isoprenaline (ISO). In these conditions, fractional shortening was increased in CHF myocytes compared to Sham (14.3 ± 1.0 vs. 9.5 ± 1.3%, p<0.05, Fig 4A and 4B). Furthermore, CHF exhibited features of contractility and Ca^2+^ handling associated with heart failure when compared to Sham: Prolonged time-to-peak of contraction (72 ± 3 vs. 48 ± 2 ms, p<0.05, Fig 4C) and prolonged time-to-half relaxation (32 ± 2 vs. 23 ± 1 ms, p<0.05, Fig 4D), as well as reduced Ca^2+^ transient amplitude (F/F_0_: 10.4 ± 0.7 vs. 14.5 ± 0.7, p<0.05, Fig 4E and 4F) and SR Ca^2+^ content (F/F_0_: 11.6 ± 1.0 vs. 14.2 ± 0.8, p<0.05, Fig 4H and 4I). The CHF group also exhibited faster rate of Ca^2+^ removal compared to Sham (24.2 ± 0.9 vs. 18.8 ± 0.4 /s, p<0.05, Fig 4G) Myocytes from MI rats exhibited preserved time-to-peak of contraction when compared to Sham (47 ± 2 vs. 48 ± 2 ms, Fig 4C), as well as unaltered time-to-half relaxation (20 ± 1 vs. 23 ± 1 ms, Fig 4D). However, Ca^2+^ transients (F/F_0_: 9.7 ± 0.7 vs. 14.5 ± 0.7, p<0.05, Fig 4F) and SR Ca^2+^ content (F/F_0_: 10.4 ± 1.0 vs. 14.2 ± 0.8, p<0.05, Fig 4I) were reduced compared to Sham.

**Fig 5:**
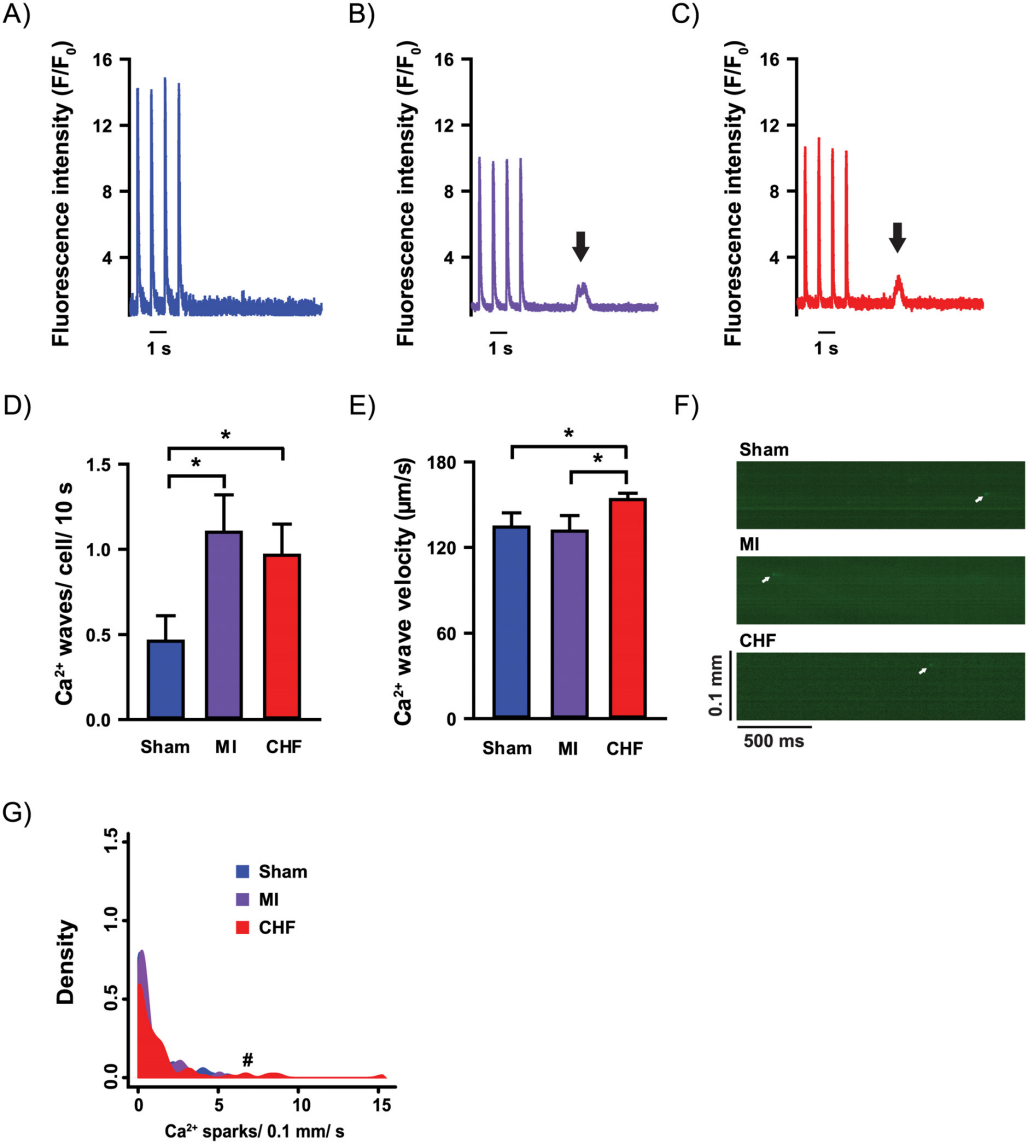
Cardiomyocyte Ca^2+^ release events under beta-adrenoceptor stimulation. Cardiomyocytes subjected to 20 nM ISO. Whole-cell Ca^2+^ imaging was used to record Ca^2+^ transients in field-stimulated cardiomyocytes and a post-stimulation rest period of 10 seconds was recorded to analyze Ca^2+^ wave (black arrows) frequency in Sham (A), MI (B) and CHF (C). Ca^2+^ wave frequency was increased in both CHF and MI compared to Sham (D), while Ca^2+^ wave velocity was increased in only CHF compared to Sham (E). White arrows illustrate Ca^2+^ sparks in confocal line-scan images (F). Increased Ca^2+^ spark frequency was found in CHF (G). n_heart_ = 3–6; n_cell_ = 9–86 for all analysis. *p<0.05 (T-test); ^#^p<0.05 (poisson test).

Interestingly, during ISO-exposure, myocytes from CHF rats exhibited a clear increase in the frequency of diastolic Ca^2+^ release events associated with arrhythmias, i.e. Ca^2+^ waves, compared to Sham (0.97 ± 0.18 vs. 0.46 ± 0.15 Ca^2+^ waves/ 10 s, p<0.05, Fig 5A–5D). Furthermore, Ca^2+^ waves in these myocytes propagated with higher velocity (154 ± 4 vs. 134 ± 10 μm/s, p<0.05, Fig 5E), indicating facilitated Ca^2+^ wave propagation. CHF myocytes also exhibited increased Ca^2+^ spark frequency compared to Sham (0.78 ± 0.27 vs. 0.63 ± 0.16 sparks/ 0.1 mm/ s, p<0.05, Fig 5F and 5G). Increased Ca^2+^ wave frequency was also present in myocytes from MI rats when compared to Sham (1.10 ± 0.22 vs. 0.46 ± 0.15 Ca^2+^ waves/ 10 s, p<0.05, Fig 5D), but contrary to CHF, Ca^2+^ spark frequency (0.65 ± 0.18 vs. 0.63 ± 0.16 sparks/ 0.1 mm/ s, Fig 5G) and Ca^2+^ wave velocity (131 ± 11 vs. 134 ± 10 μm/s, Fig 5E) was not altered in MI compared to Sham.

CHF exhibited decreased response to ISO when compared to Sham in terms of Ca^2+^ transient amplitude (155 ± 9 vs 282 ± 19% of baseline, p<0.05). The Ca^2+^ transient amplitude response to ISO was also decreased in MI compared to Sham (180 ± 11 vs. 282 ± 19% of baseline, p<0.05).

### 4. Key Ca^2+^ handling protein abundance and phosphorylation

**Fig 6:**
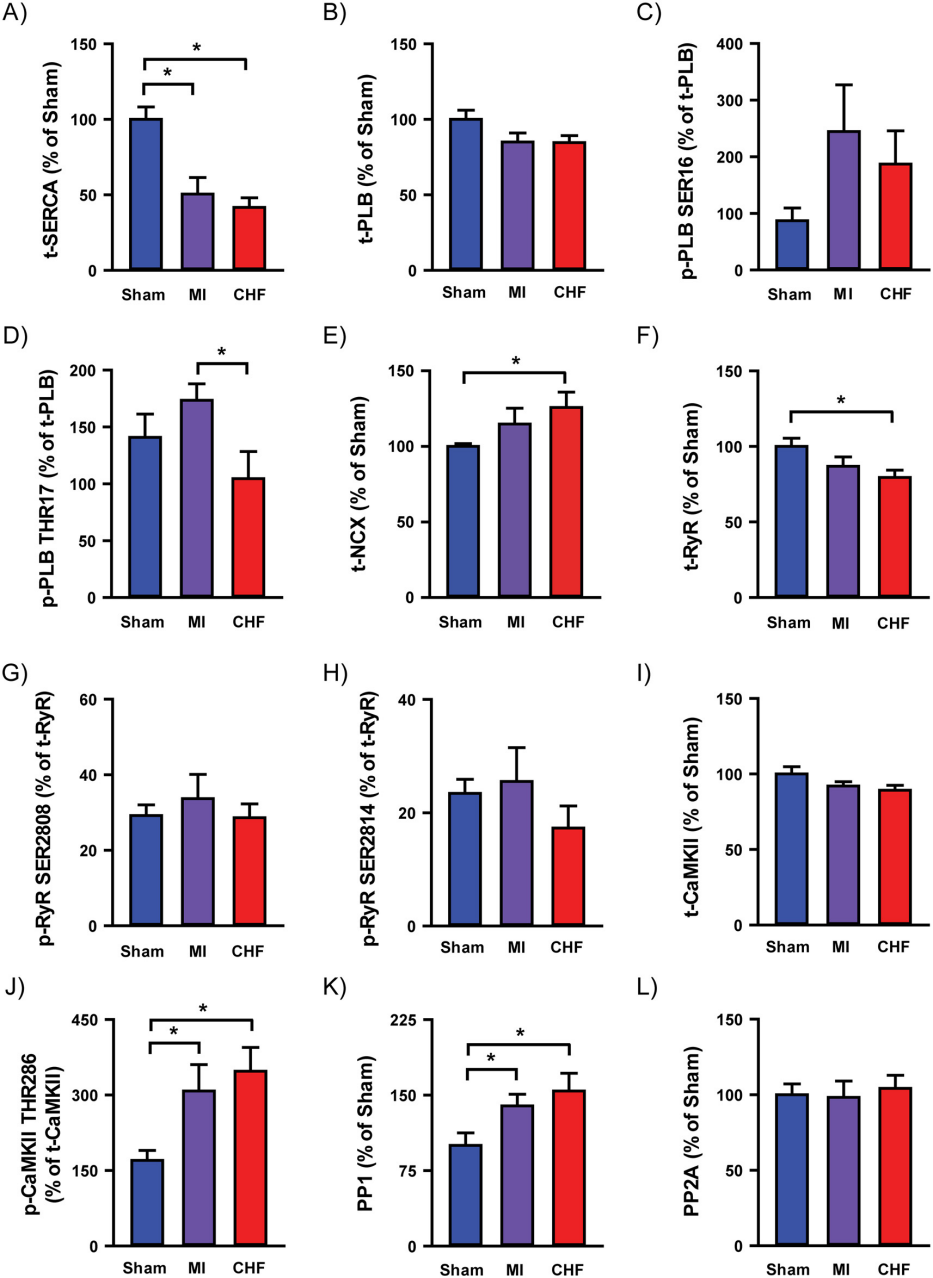
Immunoblotting. Immunoblot analysis of key Ca^2+^ handling proteins and phosphorylation was performed on tissue from left ventricles. SERCA2A abundance (A). PLB abundance (B) and phosphorylation on SER16 (C) and THR17 (D). NCX abundance (E). RyR abundance (F) with phosphorylation on SER2808 (G) and SER2814 (H). CaMKII abundance (I) and CaMKII phosphorylation on THR286 (J). PP1 (K) and PP2A (L) abundance. n_heart_ = 6 for all analysis. *p<0.05 (T-test).

Western blotting was performed to determine the abundance as well as phosphorylation of key proteins involved in cardiomyocyte Ca^2+^ handling (Fig 6 and Fig A in S1 File). In accordance with several models of heart failure, as well as human data [25], total SR Ca^2+^-ATPase (SERCA) abundance was reduced in CHF compared to Sham (42 ± 6 vs. 100 ± 8%, p<0.05, Fig 6A). Interestingly, reduced SERCA expression was also found in MI (51 ± 11 vs. 100 ± 8%, p<0.05, Fig 6A). Total phospholamban (PLB) and phosphorylated PLB were not altered in either group compared to Sham (Fig 6B–6D). CHF showed increased total Na^+^/Ca^2+^ exchanger (NCX) compared to Sham (126 ± 10 vs. 100 ± 2%, p<0.05, Fig 6E). Furthermore, the total ryanodine receptor abundance was decreased in CHF compared to Sham (80 ± 5 vs. 100 ± 6, p<0.05, Fig 6F). However, the SER2808 and SER2814 phosphorylation was not altered in CHF compared to Sham (Fig 6G and 6H). MI hearts did not express any changes in RyR abundance or phosphorylation. CaMKII and protein phosphatase 1 (PP1) are important regulators of RyR as well as PLB function. Total CaMKII abundance was unaltered in both MI and CHF compared to Sham (Fig 6I). Interestingly, CaMKII THR286 phosphorylation, an indirect indicator of CaMKII activity, was increased in both CHF and MI hearts compared to Sham (CHF 347 ± 47, MI 308 ± 53 vs. 170 ± 20%, p<0.05, Fig 6J). Furthermore, PP1 abundance was increased in both groups compared to Sham (CHF 154 ± 18, MI 139 ± 12 vs. 100 ± 13, p<0.05, Fig 6K) but no changes was found in protein phosphatase 2A (Fig 6L).

## Discussion

In this well-characterized post-infarction model of developing heart failure, myocytes from the surviving myocardium only exhibited the phenotype most often associated with heart failure after exposure to beta-adrenoceptor stimulation. With such stimulation, reduced SR Ca^2+^ content and increased Ca^2+^ wave frequency was observed compared to sham-operated controls. Interestingly, with beta-adrenoceptor stimulation, these perturbations of Ca^2+^ handling were also found in post-infarction animals without heart failure. These findings can partly explain why clinical heart failure is often revealed in situations with increased sympathetic stimulation.

### 1. The heart failure phenotype

In this post-infarction rat model of developing heart failure, myocytes from the remaining viable myocardium exhibited increased Ca^2+^ transient amplitude and fractional shortening, despite unequivocal signs of heart failure *in vivo*. These findings are contrary to several other studies of heart failure [2–7], but in accordance with previous studies of this model of developing heart failure [26, 27]. The improvement in cardiomyocyte function probably results from activation of compensatory mechanisms in the viable myocardium. Kilic *et al*. found results resembling ours in a post-myocardial infarction sheep model [28]. Two weeks after the myocardial infarction, their model exhibited significant systolic dysfunction adjacent to the infarct region, while remote regions exhibited preserved systolic function, even with a trend toward hypercontractility. Similar effects could possibly explain some of the discrepancy between cardiomyocyte function and *in vivo* cardiac function in our model too. Other studies have also found similar discrepancies between *in vivo* contractile function and *in vitro* cardiomyocyte shortening [29, 30]. These studies argue that alterations to the interstitium and cell-to-cell interactions that are lost when cells are isolated can explain such discrepancy. Furthermore, the wall-stress is altered in the failing heart and show regional heterogeneity [31, 32]. In view of these factors, the relationship between the contractile behavior of cardiomyocytes *in vivo* compared to their contractions when studied in the mechanically unloaded experimental situation might be very different for the three groups. As the focus in this study was SR Ca^2+^ handling, we have not aimed to address this further, although we do acknowledge the limitations they represent.

Our data also exemplifies the complexities of intermediate forms of ischemic heart disease and heart failure on another level: Even if we find a decreased abundance of SERCA, as expected in heart failure, the rate of Ca^2+^ removal was faster. We can only speculate that subtle alterations in the expression and function of Ca^2+^ handling proteins underlie these findings. F. ex., the increased NCX expression found in the CHF group could contribute to increased Ca^2+^ removal rate. Indeed, a previous study on the present model of developing heart failure showed both increased NCX expression and increased NCX activity [33]. The sum of subtle alterations in the expression levels of PLB, SERCA and NCX with additional post-translational modifications could combine to result in increased Ca^2+^ removal rate in CHF.

Myofilament function and beta-adrenergic response are important factors determining the contractile function of cardiomyocytes. During beta-adrenoceptor stimulation, we observed that the fractional shortening of cardiomyocytes from both post-infarction groups was preserved compared to Sham, even though the Ca^2+^ transient amplitude was decreased. One explanation for these results could be increased myofilament Ca^2+^ sensitivity [34, 35]. Reduced troponin I phosphorylation due to desensitized beta-adrenoceptors found in heart failure may be a potential explanation for an increased Ca^2+^ sensitivity [36]. Indeed, our data indicate a reduced response to beta-adrenoceptor stimulation in both MI and CHF compared to Sham, in terms of a less pronounced increase in the Ca^2+^ transient amplitude during ISO-stimulation. Our data are not suited to decipher how these different factors contribute to the resulting *in vivo* phenotype, but show that a failing phenotype cannot be easily deduced from *in vitro* findings. Indeed, even the *in vivo* findings need to be interpreted in the right context, as exemplified by our finding of decreased heart rate in CHF animal which is consistent with previous reports based on this model, whereas other reports on heart failure find varying results [22, 26, 37–39]. This could be due to differences in physiological adaptation or could represent differences in the response to anesthesia. Therefore it is important that the setting in which heart rate is recorded is consistently reported.

### 2. A destabilized RyR during beta-adrenoceptor stimulation could explain the increase in diastolic Ca^2+^ release events and depleted SR Ca^2+^ content after myocardial infarction

Hypothetically, an increased RyR open probability could explain the increased Ca^2+^ wave frequency and depletion of SR Ca^2+^ during beta-adrenoceptor stimulation [40]. CaMKII THR286 autophosphorylation, indicating increased CaMKII activation [41], was increased in both CHF and MI. CaMKII-dependent phosphorylation of RyR increases RyR Ca^2+^ sensitivity [25]. However, we did not find altered phosphorylation of either of the two most established sites for RyR-phosphorylation involved in arrhythmogenesis, i.e. SER2808 and SER2814. Furthermore, PP1 abundance was increased in both CHF and MI. Increased PP1 in CHF is in accordance with a previous study of this model of heart failure, as well as a dog model of coronary microembolization-induced heart failure, and has even been shown in myocardial tissue from patients with end-stage heart failure [42, 43]. Interestingly, Terentyev and coworkers found PP1 to increase the open probability of RyR and increasing Ca^2+^ spark frequency [44]. This was attributed to dephosphorylation of RyR. They concluded that “if phosphorylation at different sites, or sets of sites, were to affect the RyR differently, this could explain how phosphorylating and dephosphorylating agents could both lead to enhanced activity.” Increased PP1 abundance could therefore possibly contribute to destabilized RyR both in CHF and MI.

Increased open probability of RyR would increase diastolic SR Ca^2+^ leak. Such RyR-dependent leak is a potential initiator of arrhythmias by initiating Ca^2+^ waves that trigger DADs [45, 46]. In this study, we show that both failing and non-failing post-infarction rats exhibit increased Ca^2+^ wave frequency during beta-adrenoceptor stimulation even if SR Ca^2+^ content measured in a stable situation, i.e. without Ca^2+^ waves, was lower. A lower threshold for diastolic Ca^2+^ release could explain this phenomenon. The most likely explanation for a lower threshold based on existing literature and the finding of increased PP1 in this model is increased RyR sensitivity [47, 48]. Increased Ca^2+^ sensitivity of RyR could possibly also explain increased Ca^2+^ transient amplitude in CHF during baseline conditions despite unaltered SR Ca^2+^ content.

### 3. Limitations

In addition to limitations of the present study due to species-dependent differences in cardiac electrophysiology, some methodological aspects must be considered when evaluating the data. First, in theory, the cell isolation procedure could affect myocytes from CHF, MI and Sham hearts differently. We have selected cells that were considered viable based on morphological criteria and stability of Ca^2+^-dependent epifluorescence before initiation of stimulation. A potential bias in the selection of cells is therefore a problem inherent to all single cell studies. In addition, MI and CHF rats have large myocardial infarctions causing the cardiomyocyte population to be predominantly harvested from the septum, while the cardiomyocyte population from Sham animals is harvested from both the septum and the left ventricular free wall. Thus, regional differences between the septum and the free wall can affect the interpretation of the cell data. Furthermore, we have investigated cardiomyocyte function in the absence of preload or afterload. These parameters will be different in the three groups *in vivo*. Even the attachment to the laminin-coated cover slip might be different between groups [49, 50]. The discrepancy between cardiomyocyte function *in vitro* and *in vivo* could therefore potentially be different for the three groups. Lastly, our study is limited to one potential mechanism for arrhythmogenesis in heart failure, i.e. disrupted Ca^2+^ handling. It must be remembered that in patients with heart failure, as well as in animal models of heart failure, other pathological processes can also contribute to increased risk of arrhythmias, such as inflammation, fibrosis, hypertrophy, and altered connexin expression and function.

## Conclusion

This study demonstrates the coexistence of decreased SR Ca^2+^ content and increased propensity for Ca^2+^ waves in a well-characterized rat model of developing heart failure as well as in non-failing post-infarction rat hearts. However, these features were only present during beta-adrenoceptor stimulation, possibly explaining why arrhythmias in heart failure often are revealed in situations with increased sympathetic stimulation.

## Supporting Information

S1 File Antibody details and western blots. (PDF)
