# Supplementary material for: Beta-Adrenoceptor Stimulation Reveals Ca2+ Waves and Sarcoplasmic Reticulum Ca2+ Depletion in Left Ventricular Cardiomyocytes from Post-Infarction Rats with and without Heart Failure
Source: PLoS One. 2016 Apr 20;11(4):e0153887. doi: 10.1371/journal.pone.0153887 (PMC4838269; doi:10.1371/journal.pone.0153887)
Supplement: S1 File — (PDF) [file pone.0153887.s001.pdf]

Supplementary table and figure S1

Antibody details and western blots

Table A. Antibody details

Antibody target	Class	Host species	Supplier	Catalog#	Antibody ID	Final dilution
CaMKII delta	Polyclonal	Rabbit	Thermo Pierce^{TM}	PA5-22168	AB_11153337	1:10000
CamKII (phospho T286)	Polyclonal	Rabbit	Abcam	ab32678	AB_725893	1:1000
PP2A	Monoclonal	Mouse	Millipore	Cat# 05-421	AB_309726	1:2000
RyR2 Phospho Ser-2808	Polyclonal	Rabbit	Badrilla Ltd	A010-30	AB_10851615	1:5000
RyR2 Phospho Ser-2814	Polyclonal	Rabbit	Badrilla Ltd	A010-31		1:5000
PLB Phospho Ser-16	Polyclonal	Rabbit	Badrilla Ltd	A010-12		1:5000
PLB Phospho Thr-17	Polyclonal	Rabbit	Badrilla Ltd	A010-13		1:5000
PLB	Monoclonal	Mouse	Thermo Fisher Scientific Inc.	MA3-922	AB_2252716	1:5000
SERCA2 ATPase antibody	Monoclonal	Mouse	Thermo Fisher Scientific Inc.	MA3-919	AB_325502	1:2500
Cardiac NCX antibody	Polyclonal	Rabbit	GenScript	n/a	n/a	1:1000
RyR antibody	Monoclonal	Mouse	Thermo Fisher Scientific Inc.	MA3-916	AB_2183054	1:1000
PP1	Monoclonal	Mouse	Santa Cruz Biotechnology	Sc-7482	AB_628177	1:1000
Rabbit IgG, HRP-linked	Unknown	Donkey	GE Healthcare	NA934	AB_772206	1:5000
Mouse IgG, HRP-linked	Unknown	Sheep	GE Healthcare	NA931	AB_772210	1:5000

Fig A. Western blots

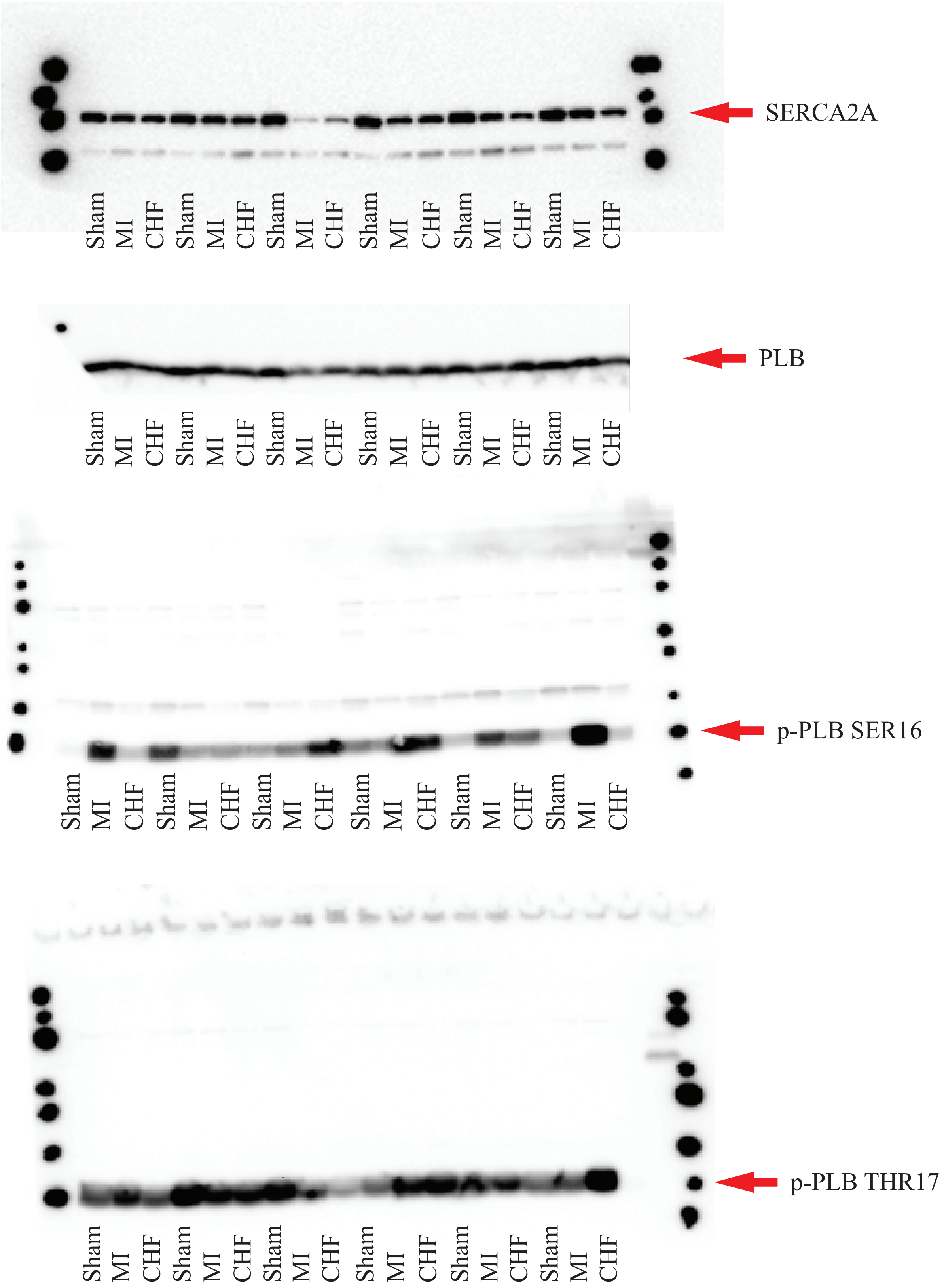

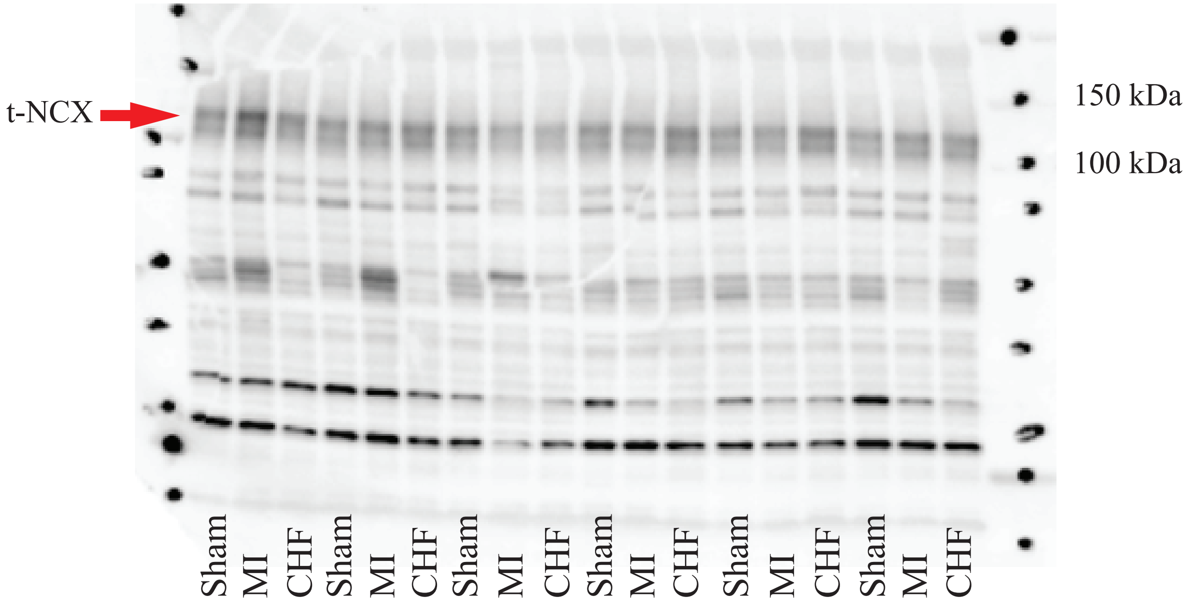

t-NCX
150 kDa
100 kDa
Sham
MI
CHF
Sham
MI
CHF
Sham
MI
CHF
Sham
MI
CHF
Sham
MI
CHF
Sham
MI
CHF

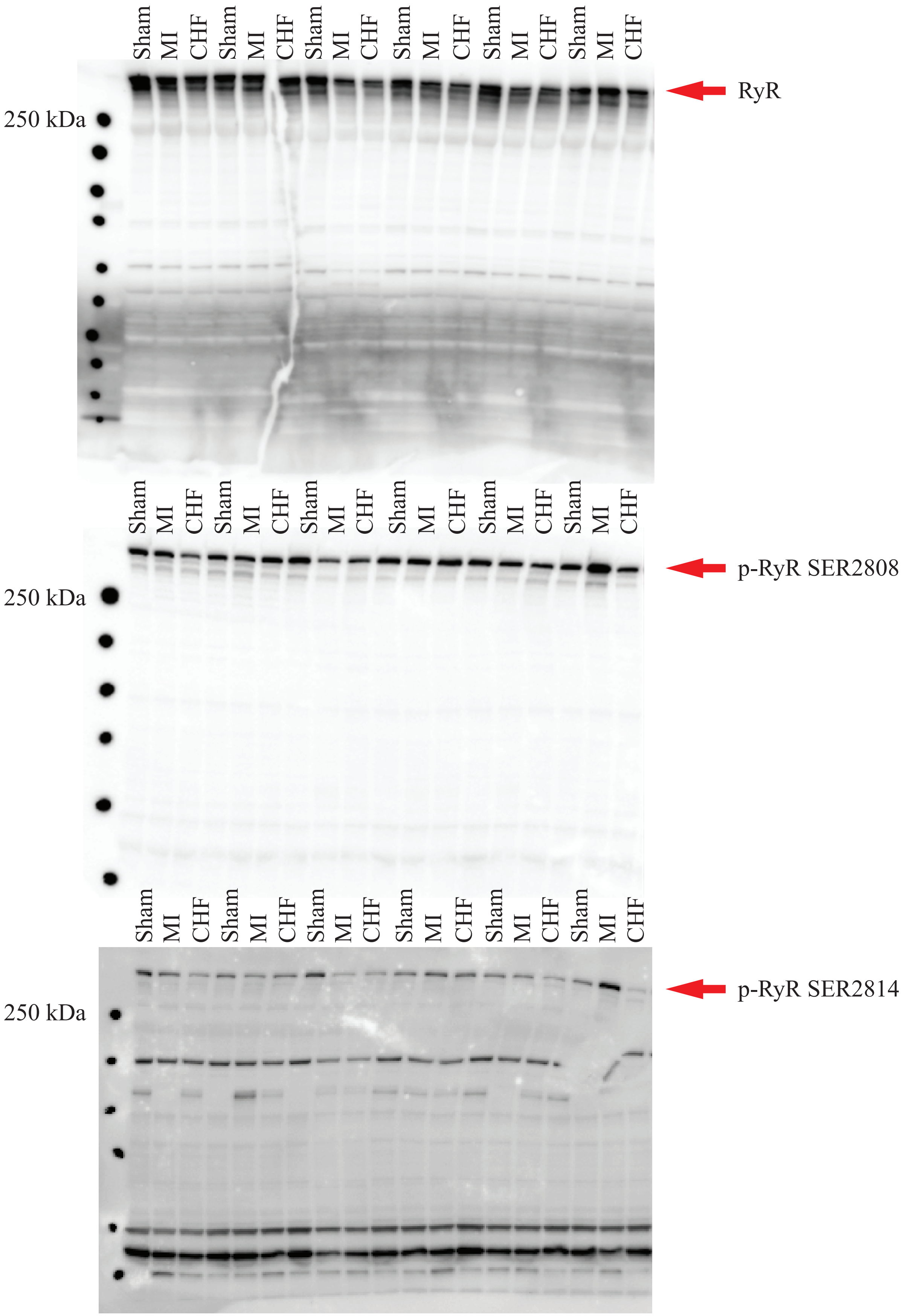
Sham MI CHF Sham MI CHF Sham MI CHF Sham MI CHF Sham MI CHF Sham MI CHF
250 kDa
RyR
Sham MI CHF Sham MI CHF Sham MI CHF Sham MI CHF Sham MI CHF Sham MI CHF
250 kDa
p-RyR SER2808
Sham MI CHF Sham MI CHF Sham MI CHF Sham MI CHF Sham MI CHF Sham MI CHF
250 kDa
p-RyR SER2814

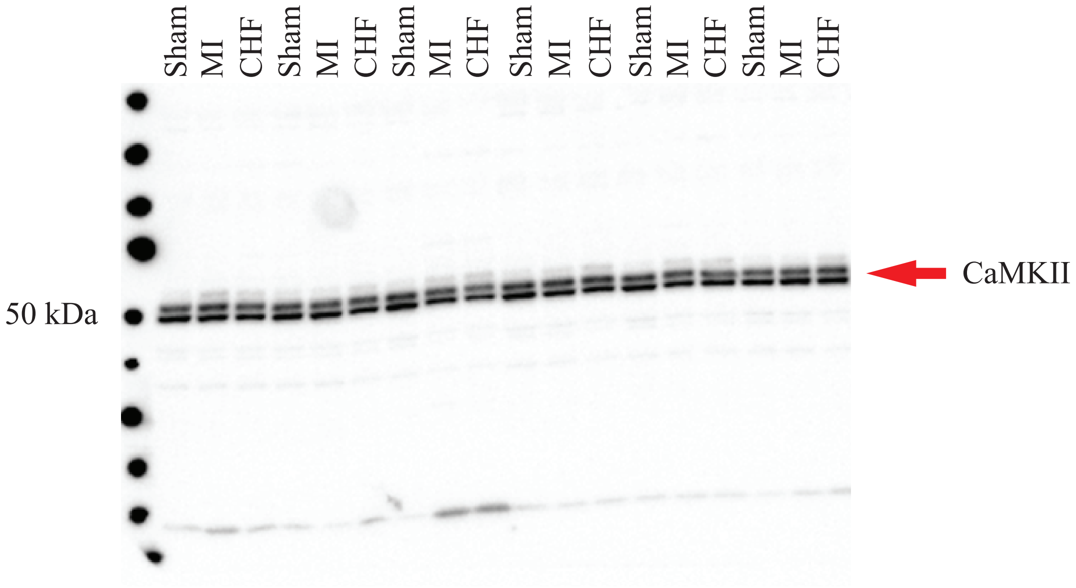
Sham
MI
CHF
Sham
MI
CHF
Sham
MI
CHF
Sham
MI
CHF
Sham
MI
CHF
Sham
MI
CHF
50 kDa
CaMKII

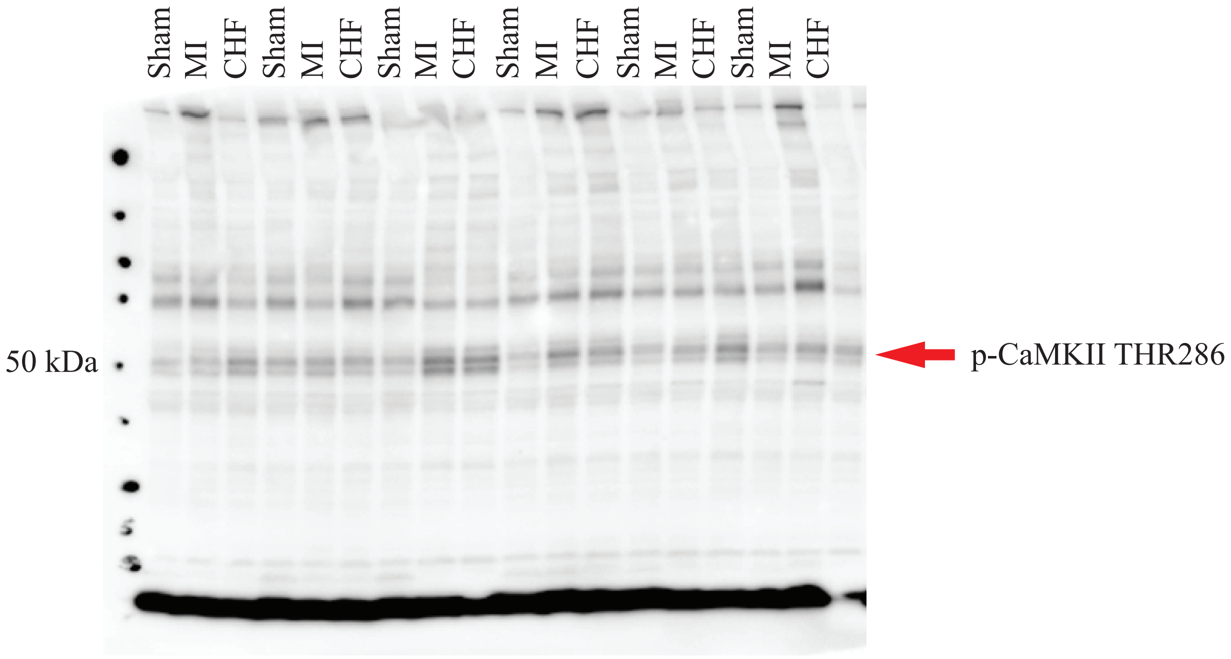
Sham
MI
CHF
Sham
MI
CHF
Sham
MI
CHF
Sham
MI
CHF
Sham
MI
CHF
Sham
MI
CHF
50 kDa
p-CaMKII THR286

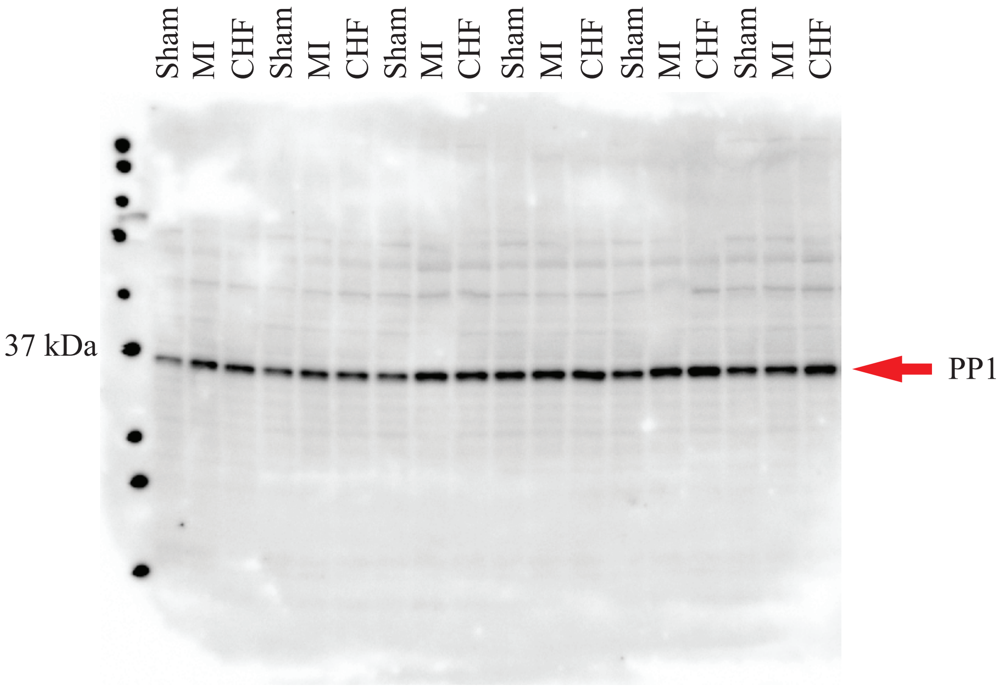
Sham MI CHF Sham MI CHF Sham MI CHF Sham MI CHF Sham MI CHF Sham MI CHF
37 kDa
PP1

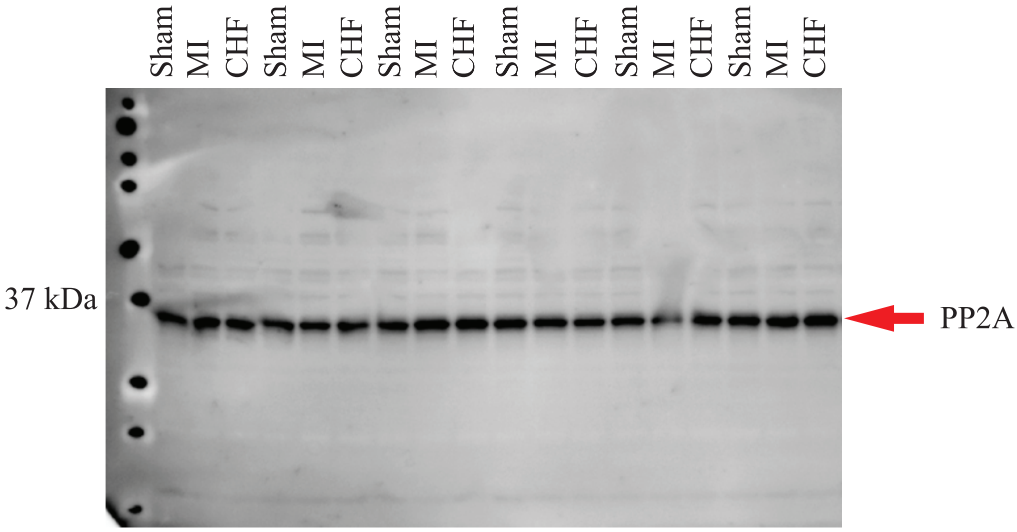
Sham MI CHF Sham MI CHF Sham MI CHF Sham MI CHF Sham MI CHF Sham MI CHF
37 kDa
PP2A
